# Assessing human-scale green equity in the 15-minute city using street-view-based visual landscape indicators and explainable machine learning: a case study of Chengdu, China

**DOI:** 10.3389/fpubh.2026.1847064

**Published:** 2026-06-08

**Authors:** Siya Yan, Yueyue Ma, Xiqian Wang

**Affiliations:** 1 College of Art and Design, Nanjing Forestry University, Nanjing, China; 2 Key Laboratory of Regional Sustainable Development Modeling, Institute of Geographic Sciences and Natural Resources Research, Chinese Academy of Sciences, Beijing, China

**Keywords:** environmental equity, semantic segmentation, street-view images, urban green space, visual landscape indicators

## Abstract

**Introduction:**

Against the backdrop of rapid urbanization, identifying disparities in green environmental exposure among different socioeconomic groups is of great importance. Taking the central urban area of Chengdu, China, as a case study, this study assesses street-level green environmental equity within a 15-minute walking distance using Baidu Street View images.

**Methods:**

A Mask2Former semantic segmentation model was employed to extract seven street-view visual landscape indicators, including Green View Index (GVI), Tree View Index (TVI), Sky View Factor (SVF), Building View Index (BVI), Hard Paving View Index (HVI), naturalness, and wildness. Second-hand housing prices were used as a proxy for socioeconomic stratification. Getis–Ord Gi*, global Moran’s I, local Moran’s I, bivariate local Moran’s I, random forest, Shapley Additive Explanations (SHAP), and partial dependence plots (PDPs) were applied to reveal the spatial relationships between housing prices and visual landscape indicators, as well as their nonlinear effects.

**Results:**

The results show that (1) housing prices in Chengdu’s central urban area exhibit significant positive spatial autocorrelation, with both high-price and low-price areas showing clear clustering patterns; (2) high-price areas generally correspond to higher TVI, GVI, and SVF values, indicating significant differences in street-view landscape exposure across housing-price groups; and (3) among the seven indicators, SVF shows strong explanatory power across all housing-price groups, whereas TVI plays a more prominent role in the high-price group.

**Discussion:**

This study deepens our understanding of green environmental equity from the perspective of street-view visual perception and provides a reference for the fine-scale optimization of urban green space allocation.

## 1 Introduction

Urbanization is a major driver of global socioeconomic transformation. According to United Nations projections, more than half of the world’s population lived in urban areas in 2019, and this proportion is expected to increase to 68% by 2050 (1). In this context, urban green space, as an important spatial carrier of urban ecosystem services, plays a vital role in improving environmental quality, alleviating ecological pressure, and promoting residents’ physical and mental health, and is widely regarded as key infrastructure for supporting inclusive and sustainable urban development (2–4). However, the value of urban green space depends not only on its total amount or scale, but also on whether its environmental benefits can be shared equitably across different social groups (5, 6). Existing studies have shown that the allocation of green resources within cities often exhibits pronounced socio-spatial disparities (2, 6, 7). Groups with higher socioeconomic status generally enjoy higher-quality green environments, whereas low-income and socially vulnerable groups have relatively limited opportunities to obtain the associated environmental benefits (8, 9). The European Commission has further noted that if green infrastructure development neglects the needs of vulnerable groups, it may exacerbate existing social inequalities (10). Therefore, examining the allocation of urban green resources and their social distributional differences from the perspective of green environment equity has become an important issue for advancing sustainable urban development and achieving Sustainable Development Goal 11 (SDG 11) under rapid urbanization.

Urban green equity is generally regarded as a specific manifestation of environmental equity in urban green space research, primarily focusing on the equitable access of different socioeconomic groups to urban green space and its associated ecological, health, and social benefits (9, 11, 12). Research on this topic has mainly examined dimensions such as green resource endowment, spatial accessibility, and exposure levels, and has revealed significant socioeconomic disparities. For example, previous studies have found systematic inequalities in access to urban green resources and spatial accessibility among different groups (13). Furthermore, Chen et al. (14) pointed out that such inequalities also extend to actual individual-level green exposure, resulting in unequal opportunities for different groups to encounter green environments and obtain potential health benefits in their daily activities.

Existing studies on urban green equity usually incorporate variables such as income, population structure, and housing prices to identify inequalities in the acquisition of green environmental benefits among different social groups (15, 16). Among these, housing prices not only reflect urban socioeconomic stratification but also, to some extent, reveal distributional differences in green environmental value after its capitalization through the housing market (17). Previous studies have shown that higher green coverage, better park proximity, and better green environmental quality are usually significantly associated with higher housing prices (18, 19). This suggests that the environmental benefits brought by green space are often capitalized in the form of housing premiums, which may further reinforce sociospatial differentiation within cities.

In assessments of urban green environments, previous studies have largely relied on top-down greening indicators to characterize the spatial distribution and accessibility of green resources, such as the normalized difference vegetation index (NDVI), green space coverage, and park accessibility (15, 16). These indicators, typically derived from remote sensing, land-use data, or spatial accessibility analysis, are useful for capturing the allocation of green resources at the regional scale, but are less capable of reflecting the green environments that residents actually encounter and perceive during walking, staying, and other daily activities along streets (20, 21). With the rapid development of street-view imagery and computer vision, a growing body of research has begun to identify visible landscape elements in street space from street-view images, thereby enabling the measurement of environmental exposure from a pedestrian perspective (22, 23). Compared with top-down indicators, street-level visual landscape indicators derived from street-view images place greater emphasis on the visibility and perceptual attributes of landscape elements from the pedestrian perspective (22, 24). By capturing multidimensional street-scale landscape features, such as greenery, sky, and buildings, these indicators better reflect residents’ actual visual experiences in daily life and have been widely applied in studies of green exposure, environmental perception, and health effects (24, 25). Against this background, this study selects a set of street-level visual landscape indicators to provide a more comprehensive characterization of the visible landscape features of street environments from the pedestrian perspective.

The 15-min city emphasizes that residents should be able to access essential functions and high-quality urban resources, including work, education, healthcare, commerce, and leisure, within a daily living circle reachable by walking or cycling. This provides an important spatial analytical framework for examining green equity at the human scale (26, 27). Under this framework, green equity should not be understood merely as the balanced allocation of green resources at the macro spatial level, but should also pay attention to residents’ actual accessibility to green environments within their daily living circles, as well as differences in environmental exposure and perceptual experience during walking and everyday activities (22, 28). However, to more systematically reveal the potentially nonlinear relationships between perceived green characteristics and housing prices, as well as their spatial coupling mechanisms, this study introduces street-view visual landscape indicators within the 15-min city framework and combines them with interpretable machine learning methods to investigate how green perception in street space influences housing prices and the green equity issues reflected therein.

This study takes the central urban area of Chengdu as the study area and introduces the 15-min city framework to examine the relationship between street green environments and housing prices from the perspective of residents’ daily walking. On this basis, street-view images are used to measure visible street greenery, and housing prices are incorporated to explore the capitalization effect of green environmental value and its spatial distributional differences. The main contributions of this study are threefold. First, at the indicator construction level, this study develops a street-level visual landscape analytical framework based on street-view images, extracts seven types of visual landscape indicators, and aggregates them at the 15-min walking scale to characterize community-level street green environment exposure from the perspective of residents’ perception, thereby extending the measurement of street-scale green environmental quality. Second, at the analytical framework level, this study integrates spatial statistical analysis and interpretable machine learning methods to depict the spatial differentiation pattern and clustering characteristics of housing prices in the central urban area of Chengdu, and to reveal the spatial association between visual landscape indicators and housing prices, thereby extending research on street-view perceived environments to the explanatory dimension of housing value differentiation. Third, at the level of empirical findings and practical implications, this study finds that the effects of street-view landscape factors vary substantially across different housing price levels, exhibiting clear stratification and heterogeneity. These findings provide empirical evidence for optimizing green infrastructure and informing environment equity-oriented urban planning interventions in the context of rapid urban expansion.

## 2 Datasets and study area

### 2.1 Study area

**Figure 1:**
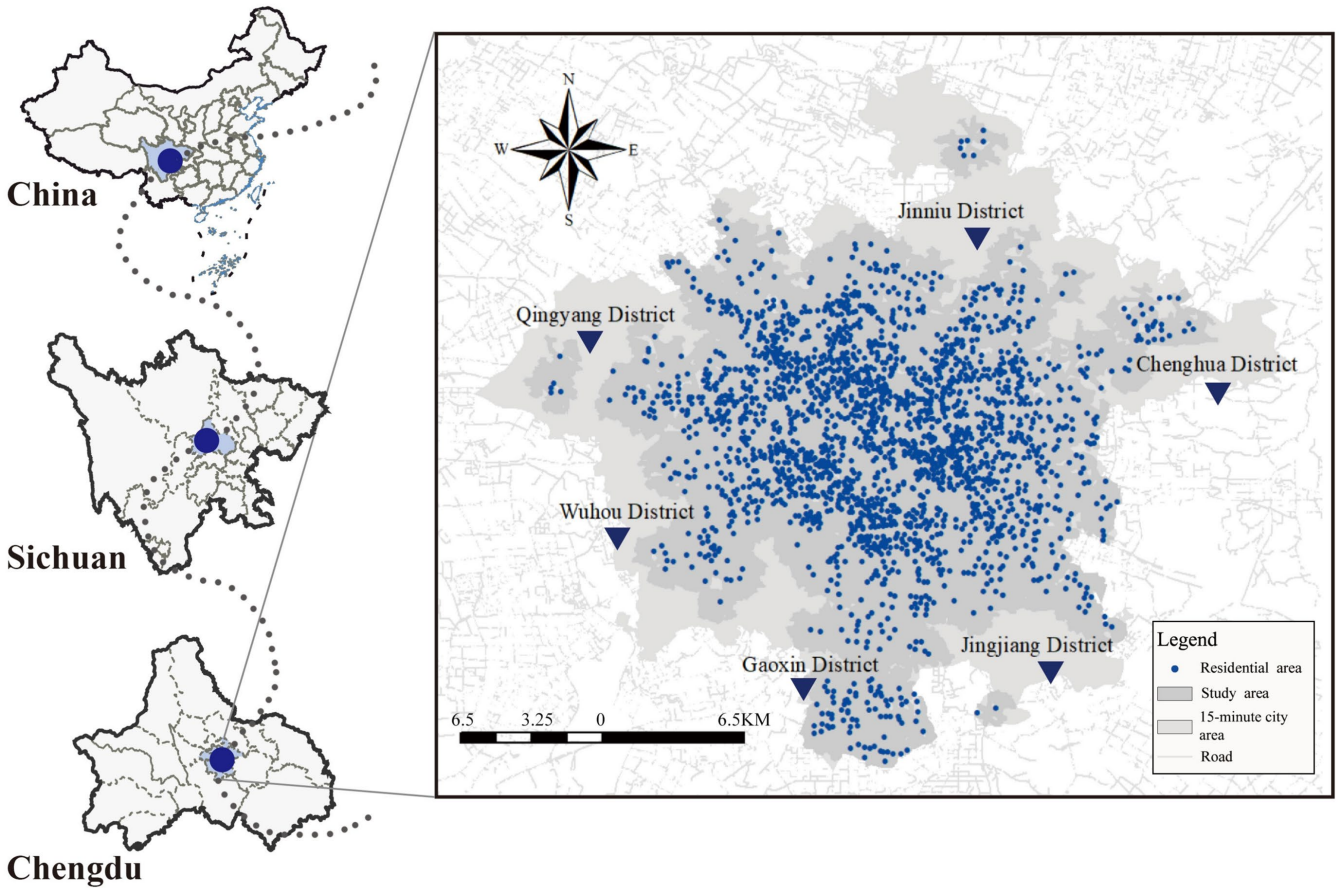
Overview of the study area.

Chengdu, located in southwestern China, is a national central city and the core of the Chengdu–Chongqing dual-city economic circle. This study selects six major administrative districts within Chengdu’s central urban area—Jinniu District, Chenghua District, Jinjiang District, Wuhou District, Qingyang District, and Chengdu Hi-Tech Zone—as the study area, based on two key considerations. First, this region concentrates the highest population density and core urban functions, representing typical urban social structures and spatial characteristics. According to the Seventh National Population Census (2020) (29), these districts exhibit population densities significantly higher than the citywide average, with some exceeding 17,000 people per km^2^. Second, despite advancements in green infrastructure, Chengdu’s central districts still experience limited green space availability and uneven spatial distribution. As of 2022, the built-up area of the city reported a green coverage rate of 39.5%, a green space ratio of 35.2%, and per capita park green space of 12.1 m^2^ (30)—below the United Nations’ recommended threshold of 15 m^2^ per person for livable cities. In recent years, Chengdu has demonstrated strong policy momentum by initiating and implementing the “Park City” strategy, underscoring the government’s emphasis on equitable access to green resources. This initiative is closely aligned with the United Nations Sustainable Development Goals (SDGs), particularly Goal 11 (Sustainable Cities and Communities) and Goal 3 (Good Health and Well-being), reflecting a commitment to inclusive, health-oriented urban development (Figure 1).

### 2.2 Datasets

This study utilized second-hand housing transaction price data obtained from the Beike real estate platform,^1^ representing residential transactions that occurred in 2023. The dataset comprises 25,242 geolocated records of residential communities. To ensure sample consistency, only one transaction price record per community was manually retained, as multiple listings may exist within the same residential area. Administrative boundary data were sourced from the National Geoinformation Public Service Platform,^2^ while road network data were acquired from Amap^3^ as of 2023. Additionally, street-view images were collected in 2023 using a customized Python script to retrieve panoramic imagery from Baidu Maps (Table 1).^4^

**Table 1:** Summary of datasets used in this study.

Data type	Data source	Description
Roads	Amap (https://ditu.amap.com/)	Road network data for Chengdu as of June 2023, including highways, arterial roads, sidewalks, and roads of different classes.
Administrative boundaries	National Geographic Information Public Service Platform (https://www.tianditu.gov.cn/)	Including provincial, municipal, and county-level administrative divisions.
House prices	Beike platform (https://ke.com/)	Including information on the number, price, address, latitude, and longitude of second-hand housing units in Chengdu’s urban area in 2023.
Street view image	Baidu Maps (https://map.baidu.com/)	Street-view images were collected from Baidu Maps using customized Python scripts in July 2023.

## 3 Methodology and methods

### 3.1 Methodological framework

**Figure 2:**
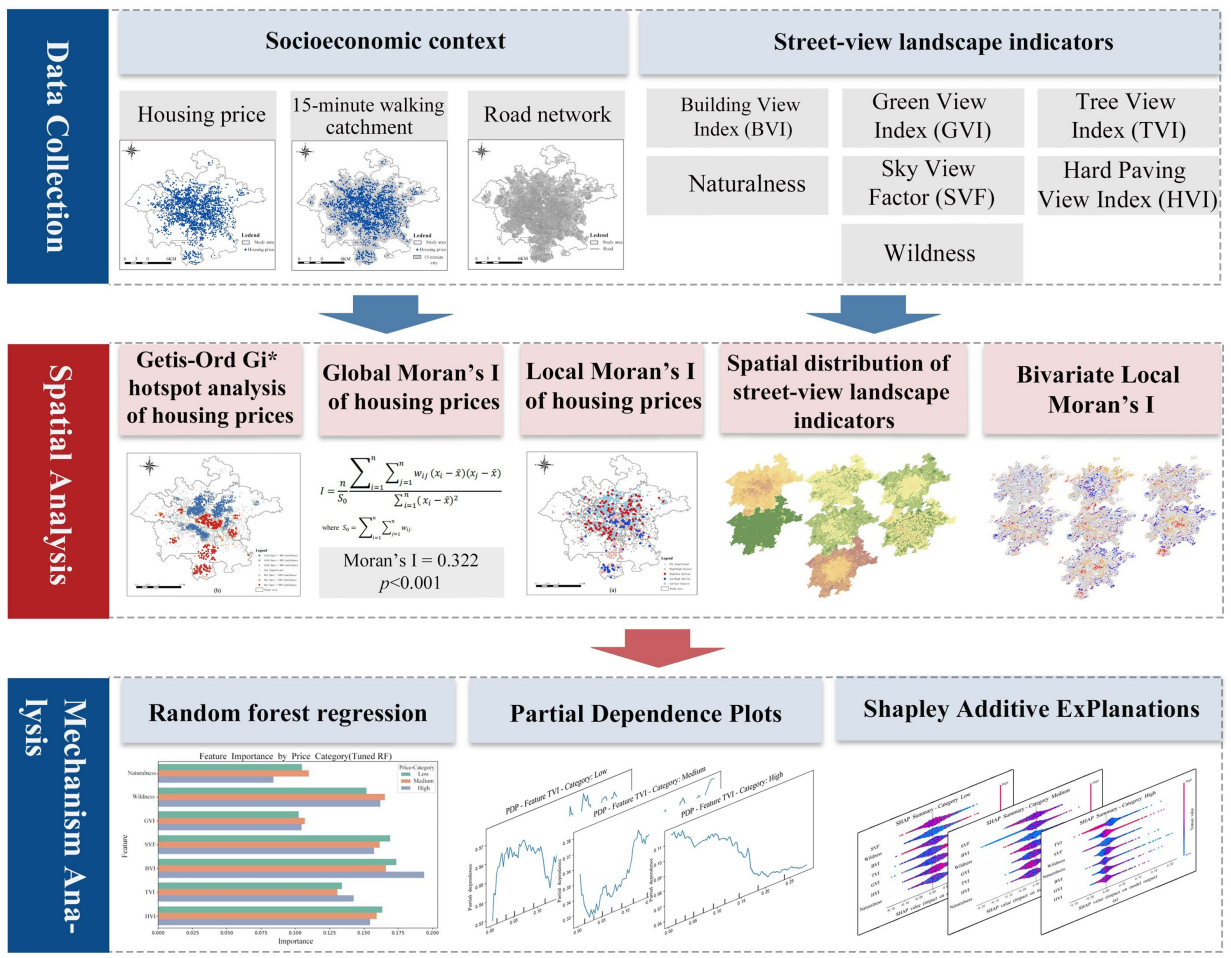
Technical route.

Figure 2 presents the overall analytical framework of this study, which comprises three stages: data collection, spatial analysis, and model interpretation. In the data collection stage, an integrated dataset was constructed by combining housing prices, 15-min walking catchments, road networks, and visual landscape indicators, including the Building View Index (BVI), Green View Index (GVI), Tree View Index (TVI), Sky View Factor (SVF), Hard Paving View Index (HVI), Naturalness, and Wildness. In the spatial analysis stage, Getis–Ord Gi* hotspot analysis, global Moran’s I, and local Moran’s I were applied to analyze the spatial clustering characteristics of housing prices. In addition, the spatial distribution patterns of street-view landscape indicators were examined, and bivariate local Moran’s I was used to identify the local spatial associations between housing prices and street-view landscape indicators. In the model interpretation stage, a random forest regression model was developed and combined with Partial Dependence Plots (PDPs) and Shapley Additive Explanations (SHAP) to reveal the relative importance, effect direction, and heterogeneous influences of different street-view landscape indicators on housing price disparities.

### 3.2 Street-view image acquisition based on the 15-minute city framework

In this study, residential communities involved in second-hand housing transactions were used as the basic spatial unit, and a walkable street network was constructed using ArcGIS network analysis tools. Based on an average walking speed of 80 meters per minute, a 15-min walking time threshold was set to delineate the accessible area around each community, thereby defining the spatial boundary of the “15-Minute City.” Compared to traditional Euclidean buffers, service areas derived from road network analysis more accurately reflect the built environment experienced by residents during daily travel (31). Along the street network, sampling points were placed at 100-meter intervals to simulate residents’ visual perception of surrounding green elements during walking. A spatial linkage method was applied to associate each sampled street view point with the residential community located within the corresponding 15-min service area, ensuring that the collected data accurately represent the urban landscape features encountered in everyday life.

A total of 25,242 sampling points were generated, and 19,630 panoramic street-view images were collected through the Baidu Street View (BSV) platform, with each point providing a 360-degree perspective. These images were semantically segmented using the Mask2Former model, which was trained on the ADE20K dataset (32). Compared to widely used models such as PSPNet (33), UNet (34), and DeepLabv3 (35), Mask2Former integrates a mask attention mechanism with multi-scale feature representation, offering superior contextual modeling capabilities and segmentation performance in complex urban scenes (36). The model focused on extracting key urban landscape elements, including greenery, trees, sky, and buildings, thereby providing a robust image-based foundation for the subsequent analysis of green space distribution and environmental equity.

### 3.3 Construction of the indicator system

To systematically quantify the multidimensional visual characteristics of the urban street environment, this study employed Street View Imagery (SVI) in combination with computer vision methods to measure environmental perception at the street scale. Previous studies have shown that street-view imagery can capture street environmental characteristics relatively realistically from the pedestrian perspective and has been widely applied in urban analysis and spatial perception research (24). Furthermore, Zhang et al. (37) pointed out that, with the support of artificial intelligence and computer vision techniques, SVI can be used to automatically extract multidimensional visual features at the street-level over large areas, thereby providing reliable support for the refined quantification of the urban visual environment.

It should be noted that greenery perception in streets is not entirely equivalent to the amount of green pixels within the visible field. Previous research has shown that although GVI is one of the most commonly used indicators for measuring street-level greenery, it essentially reflects the visibility of vegetation elements from the pedestrian perspective (38). However, the street-view experience is not determined solely by visible greenery, but is jointly shaped by multiple visual components, including green-view characteristics, spatial form, and other street-interface elements (39).

Based on this, this study constructed a street-view visual indicator system comprising three categories: green indicators, perceptual indicators, and supplementary indicators. Specifically, GVI was used to represent the overall proportion of visible vegetation. Considering the particular value of street trees in environmental improvement and green equity (40), this study further introduced TVI to distinguish trees from general vegetation, thereby reflecting the roles of canopy shading and tree-canopy visibility. Naturalness and Wildness were used to supplement the characterization of the perceived natural attributes of street -views, thereby reflecting differences at the level of subjective perception. BVI and SVF, respectively, represent the visual proportion of the building interface and the degree of openness of street space. Among them, SVF, by measuring the proportion of visible sky from the pedestrian perspective, characterizes the openness and enclosure of street space and compensates for the limitations of a single greenery indicator from the perspective of spatial morphology (41, 42). HVI was used to characterize the visual proportion of hard surfaces, such as roads and paving, in street -views, reflecting the non-green visual components corresponding to green elements and thus providing supplementary information for identifying the environmental context in which green exposure is embedded (Table 2).

**Table 2:** Descriptions of the panorama-based metrics used in this study.

Metrics	Descriptions of metrics	Complementary explanation
Green view index (GVI)	The cumulative ratio of all greenery elements	Indicates the overall visible greenness in street -view images and represents pedestrians’ visual exposure to street -greenery.
Building view index (BVI)	Proportion of the buildings	Indicates the visible proportion of buildings and reflects the dominance of the built environment and the degree of spatial enclosure.
Hard paving view index (HVI)	The proportion of visibility of hard pavement in the picture	Indicates the visible proportion of roads, sidewalks, and other hard-paved surfaces, reflecting the visual exposure to grey space.
Sky view factor (SVF)	The proportion of visibility of the sky area in the image	Indicates the visible proportion of the sky and reflects the openness of street space and the degree of view obstruction by buildings or tree canopies.
Tree view index (TVI)	The proportion of visibility of tree areas in the image.	Indicates the visible proportion of trees and reflects visual exposure to tree canopy greenness.
Naturalness	The arctangent of the ratio of natural elements (e.g., greenery, water, rock, animals, and mountains) to grey infrastructure (e.g., buildings, pavement, natural elements and roads). naturalness= $\arctan(\frac{\left[\text{natural elements}\right]}{\left[\text{grey infrastructure}\right]})$ (58)	Reflects the overall dominance of natural elements over grey infrastructure in the street -view environment.
Wildness	The arctangent of the ratio of flora to the sum of grey infrastructure, artificial facilities (benches, lampposts, etc.), and grass. Wildness= $\arctan(\frac{\left[\text{flora}\right]}{\left[\text{grey infrastructure}]+[\text{facility}]+[\text{grass}\right]})$ (58)	Reflects the degree to which the street -view environment appears less managed and more spontaneously natural.

### 3.4 Spatial autocorrelation analysis

To preliminarily assess whether housing prices exhibit significant spatial clustering, supplementary spatial clustering analyses were conducted using hot spot analysis (Getis–Ord Gi*), Global Moran’s I, and Local Moran’s I. Detailed methods and formulae are provided in the Supplementary material S1. The results indicate that housing prices in the study area exhibit significant spatial autocorrelation and local clustering, thereby providing contextual support for the subsequent analysis of the local spatial coupling between housing prices and visual landscape indicators.

#### 3.4.1 Bivariate local Moran’s I

Bivariate Local Moran’s I was used to analyze the local spatial associations between housing prices and street-view visual landscape indicators (43, 44). The statistic is calculated as shown in Equation (1):


$$I_{i}^{\mathrm{xy}}=z_{x}^{i}\sum_{j=1}^{n}w_{\mathrm{ij}}z_{y}^{j}$$ (1)

The local spatial association patterns can be classified into four types: high–high (HH), low–low (LL), high–low (HL), and low–high (LH). Among them, HH and LL indicate local spatial coupling, whereas HL and LH indicate local spatial mismatch.

### 3.5 Random forest regression

To investigate the effects of visual landscape indicators on housing prices, this study constructed a Random Forest Regression (RFR) model, using seven visual landscape indicators as explanatory variables and housing prices as the response variable. This method demonstrates high flexibility and generalization capability in modeling nonlinear, high-dimensional, and multi-variable interactive data (45), and has been widely applied in urban spatial process simulation and real estate valuation research (46, 47). Random forest is an ensemble learning method that constructs multiple decision trees based on different subsets of training samples and feature variables, and integrates their predictions through weighted averaging to improve model accuracy and generalization (45). In the regression setting, the model output is expressed as the average of predictions from all individual trees, where $h_{t}(x)$ denotes the prediction of the t -th regression tree for input sample x . The regression output can be expressed as Equation (2):


$$\hat{f}(x)=\frac{1}{T}\sum_{t=1}^{T}h_{t}(x)$$ (2)

### 3.6 Interpretability

As random forest is considered a “black-box” model, this study further introduced two mainstream model interpretability techniques—PDP and SHAP—to reveal the internal variable influence mechanisms and marginal contributions within the model.

#### 3.6.1 Partial dependence plot

PDP estimates the marginal effect of a target feature $x_{s}$ on the model prediction by averaging the predicted responses over the joint distribution of all other features $x_{C}$ , while keeping $x_{s}$ fixed at different values. This technique helps to characterize the model’s dependence on $x_{s}$ by isolating its influence from that of other variables (48). Mathematically, this is defined as Equation (3):


$${\hat{f}}_{x_{s}}(x_{s})=E_{x_{C}}[\hat{f}(x_{s},x_{C})]=\int \hat{f}(x_{s},x_{C})\;\mathrm{dP}(x_{C})$$ (3)

where $x_{s}$ denotes the target feature, $x_{C}$ represents all other features, and $\hat{f}$ is the trained prediction function. The integral reflects the marginalization of the model output over the joint distribution of $x_{C}$ . In this study, PDP curves were generated for each landscape variable across different housing price segments to identify their nonlinear response patterns and value-sensitive intervals.

#### 3.6.2 SHAP

To further interpret model outputs at the individual sample level, this study introduced the SHAP method. Previous studies (49, 50) have demonstrated that SHAP provides strong explanatory power and effective visualization in real estate valuation, urban landscape quality assessment, and spatial equity analysis. Based on the Shapley value theory from cooperative game theory, SHAP quantifies the marginal contribution of each input feature to a single prediction, offering both consistency and additive interpretability (51). The additive form of the SHAP model is defined in Equation (4):


$$\phi_{i}=\sum_{S\subseteq N\setminus \{i\}}\frac{|S|!(|N|-|S|-1)!}{|N|!}[f_{S\cup \{i\}}(x_{S\cup \{i\}})-f_{S}(x_{S})]$$ (4)

where $\phi_{i}$ denotes the SHAP value of feature i , $f_{S}$ refers to the model trained on a subset of features S , and N is the full set of input features. In this study, the average SHAP values of all landscape variables were computed to identify the most influential landscape elements on housing prices under the context of the “15-min city.”

## 4 Results

Supplementary spatial analyses showed significant spatial autocorrelation and local clustering in housing prices across the study area (see Supplementary Figures S1, S2; Supplementary Table S1). High-value clusters were mainly concentrated in the southern part of the study area and several central urban districts, whereas low-value clusters were primarily located in the northern and peripheral areas, indicating a clear non-random spatial distribution of housing prices. These findings provided essential contextual support for the subsequent analysis of the spatial patterns of visual landscape indicators and their relationships with housing prices.

### 4.1 Spatial distribution of visual landscape indicators

**Figure 3:**
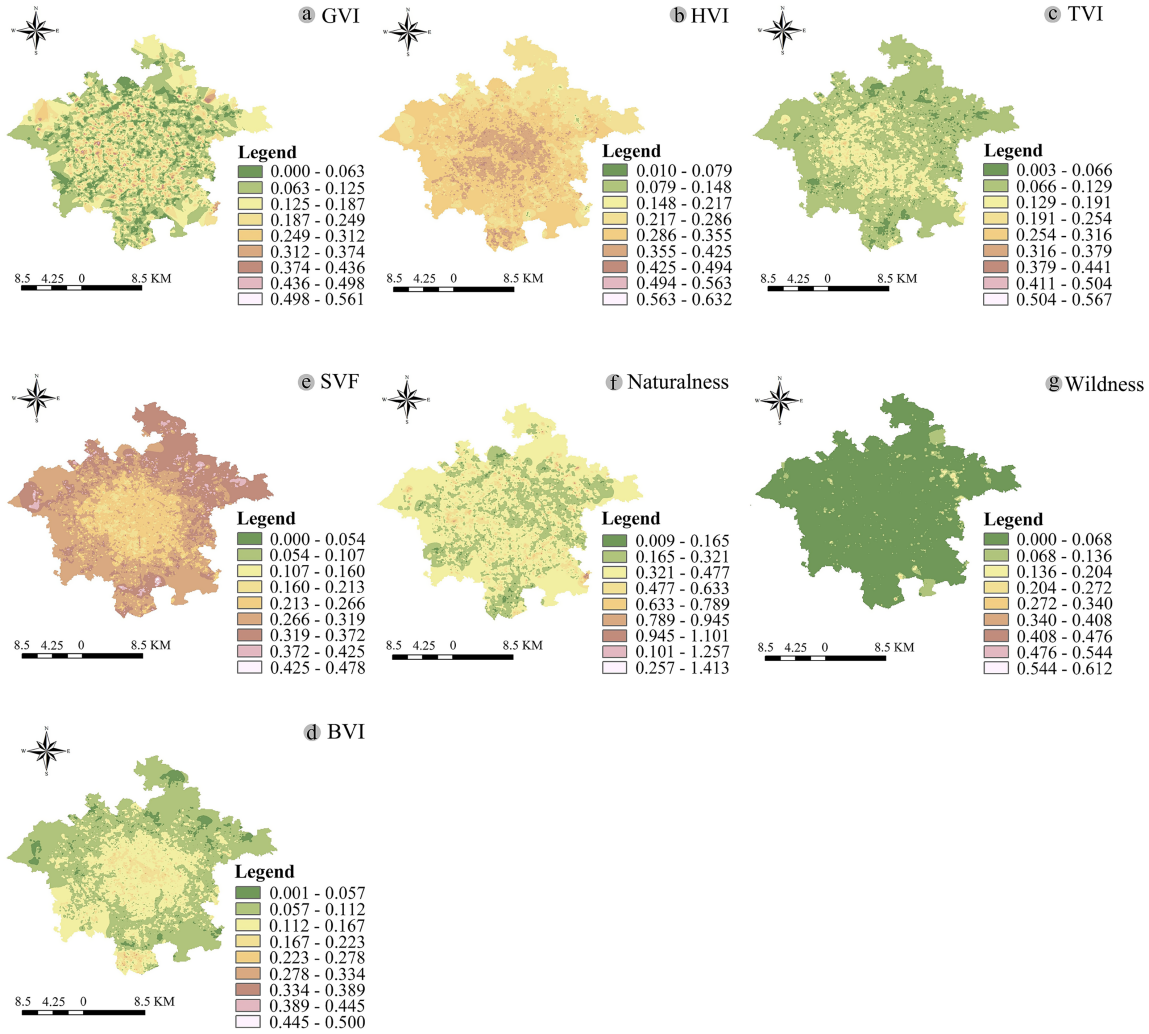
Spatial distribution of visual landscape indicators.

As shown in Figure 3, different visual landscape indicators exhibited pronounced spatial heterogeneity across the study area. Green perception-related indicators and naturalness-related indicators generally tended to show higher levels in peripheral areas, whereas indicators related to spatial openness and hardscape exposure displayed different localized variation patterns. Overall, GVI, TVI, Naturalness, and BVI showed similar spatial patterns, with relatively higher values in the peripheral parts of the study area and lower values in the central built-up area.

Specifically, GVI showed relatively high levels in urban fringe areas and some outer districts, whereas the inner urban area was dominated by low to medium values. TVI exhibited a similar trend, with high-value areas more frequently distributed in peripheral areas and some southern districts. Naturalness was generally higher in outer areas, while BVI showed a typical pattern of lower values in the center and higher values in the periphery. These spatial patterns may be associated with systematic differences in built-environment morphology and landscape configuration between the central urban area and peripheral zones. In the central built-up area, higher building density, stronger street enclosure, and the continuous distribution of artificial surfaces likely constrained the spatial expression of GVI, tree-canopy exposure, and naturalness-related indicators. By contrast, peripheral areas were characterized by lower development intensity and more abundant open spaces, adjacent green spaces, and roadside greenery, which were more conducive to higher levels of green perception and naturalness.

By contrast, SVF and HVI exhibited another type of spatial pattern. SVF was generally higher in peripheral areas and lower in the central urban area, indicating that street spaces in the central built-up area were more compact and that building enclosure imposed stronger constraints on sky visibility. Peripheral areas, with greater spatial openness, usually had better ventilation, daylight access, and visual extensibility. HVI was generally dominated by low to medium values, but was relatively higher in some parts of the central urban area, suggesting that hardscape interfaces and artificial surface exposure were more common in high-density built environments. This pattern was consistent with the continuous building interfaces and the higher degree of surface artificialization in the central urban area.

Wildness showed the most distinctive spatial distribution, with low values across most of the study area and relatively high values appearing only in a few localized areas. This indicated that the street environment in central Chengdu was still predominantly characterized by highly managed urban landscapes with strong human intervention, whereas spatial units with stronger natural wildness were relatively limited. Although some areas exhibited a certain degree of greening and natural attribute exposure, the overall landscape pattern of the study area remained dominated by a highly managed and built-up urban environment, with wildness preserved only in a few marginal locations.

### 4.2 Bivariate local Moran’s I analysis between housing prices and visual landscape indicators

**Figure 4:**
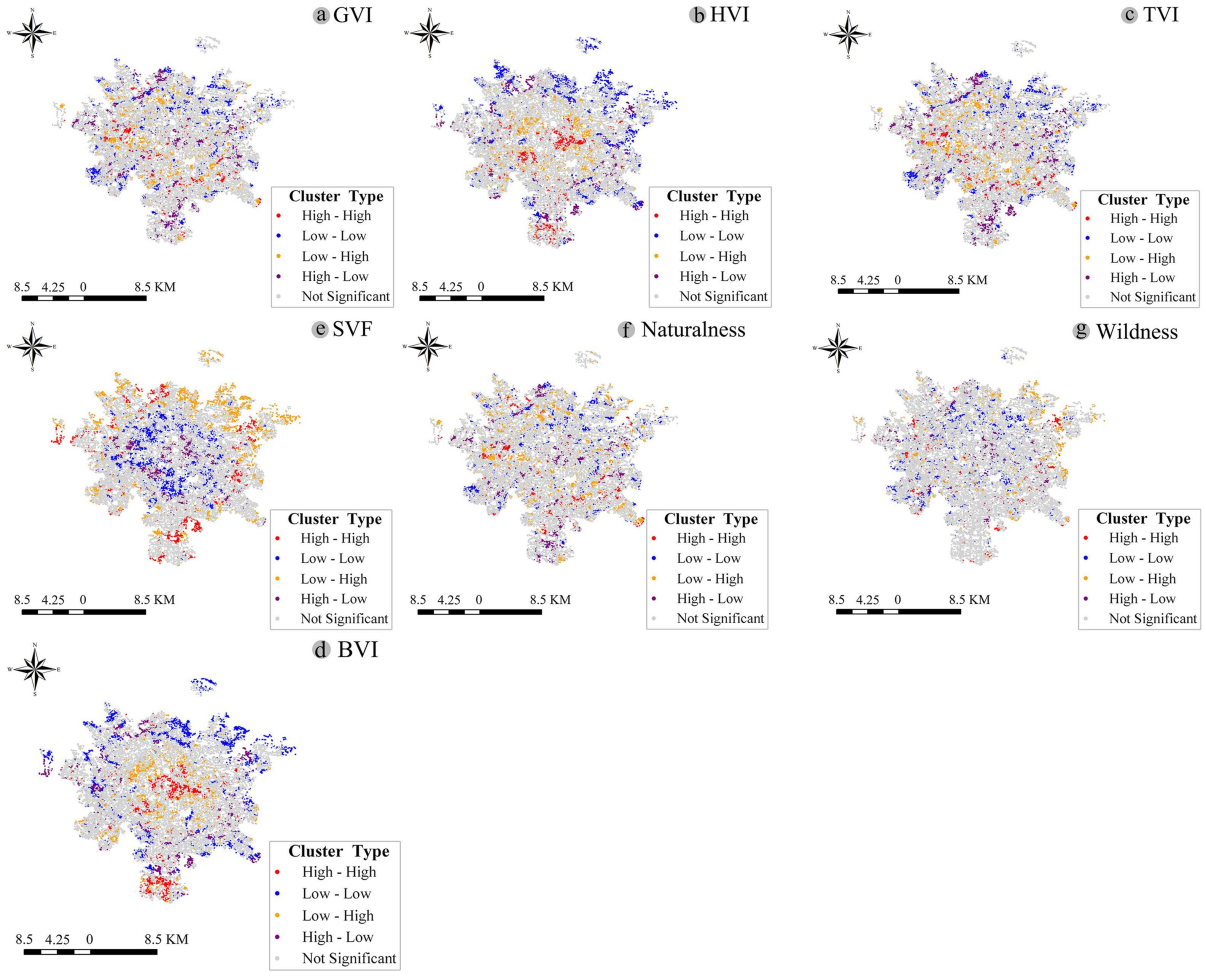
Bivariate local Moran’s *I* cluster maps between housing prices and visual landscape indicators.

As shown in Figure 4, all visual landscape indicators exhibited some degree of local spatial association with housing prices, but significant differences were observed in cluster type, spatial location, and clustering intensity, indicating that high-priced residential spaces did not form consistent spatial coupling with all landscape characteristics. Overall, several indicators showed pronounced high–high (HH) clusters in the high-price areas of the southern part of the study area. Combined with the housing price hot spot analysis presented in the Supplementary material, the high-price areas in central Chengdu were mainly concentrated in the southern parts of Gaoxin District, Wuhou District, and Jinjiang District. Therefore, the co-occurrence of high values for multiple visual landscape indicators in these areas reflected a strong local spatial coupling between high housing prices and specific street-environment characteristics.

Among them, the HH clusters between TVI and housing prices were the most pronounced, mainly distributed in the central-southern part of the study area, particularly in Gaoxin District and its adjacent areas. BVI also showed relatively evident HH clusters with housing prices, and these co-clustered high-value areas were likewise mainly located in the southern high-price zones. The HH clusters between SVF and housing prices were also prominent in the southern area, with additional localized high-value co-clustering in some peripheral zones. These results suggested that street environments characterized by higher tree coverage, stronger building interface features, and greater spatial openness were more likely to co-occur with high-priced residential units.

By contrast, although GVI, Naturalness, and Wildness also showed HH clusters with housing prices, their distributions were more scattered and localized overall. Specifically, the HH clusters of GVI and Naturalness were mainly scattered across the central-southern and some peripheral areas, indicating that high-priced areas showed a certain preference for street environments with higher visible greenness and stronger natural attributes, although this relationship had not formed a large-scale and spatially continuous clustering pattern. The number of HH clusters between Wildness and housing prices was relatively limited and was observed only in a few marginal locations, indicating that natural landscapes with stronger wildness were not a common feature of high-priced spaces in the central urban area. Meanwhile, the local spatial relationship between HVI and housing prices was relatively complex. Although HH clusters were also found in the southern part of the study area, low–low (LL) clusters and some outlier units were also common, indicating that hardscape exposure did not show a unidirectional spatial correspondence with housing prices but instead exhibited stronger spatial heterogeneity.

Overall, significant spatial coupling existed between high-priced areas in central Chengdu and some visual landscape indicators. Among them, the high-value co-clustering of TVI and SVF with housing prices was more prominent, whereas the associations of GVI, Naturalness, and Wildness with housing prices were relatively scattered, and HVI showed a more complex pattern of local spatial correspondence. These findings indicated clear differences in the local spatial associations between different visual landscape indicators and housing prices.

### 4.3 Relative importance of visual landscape indicators across housing price levels

**Figure 5:**
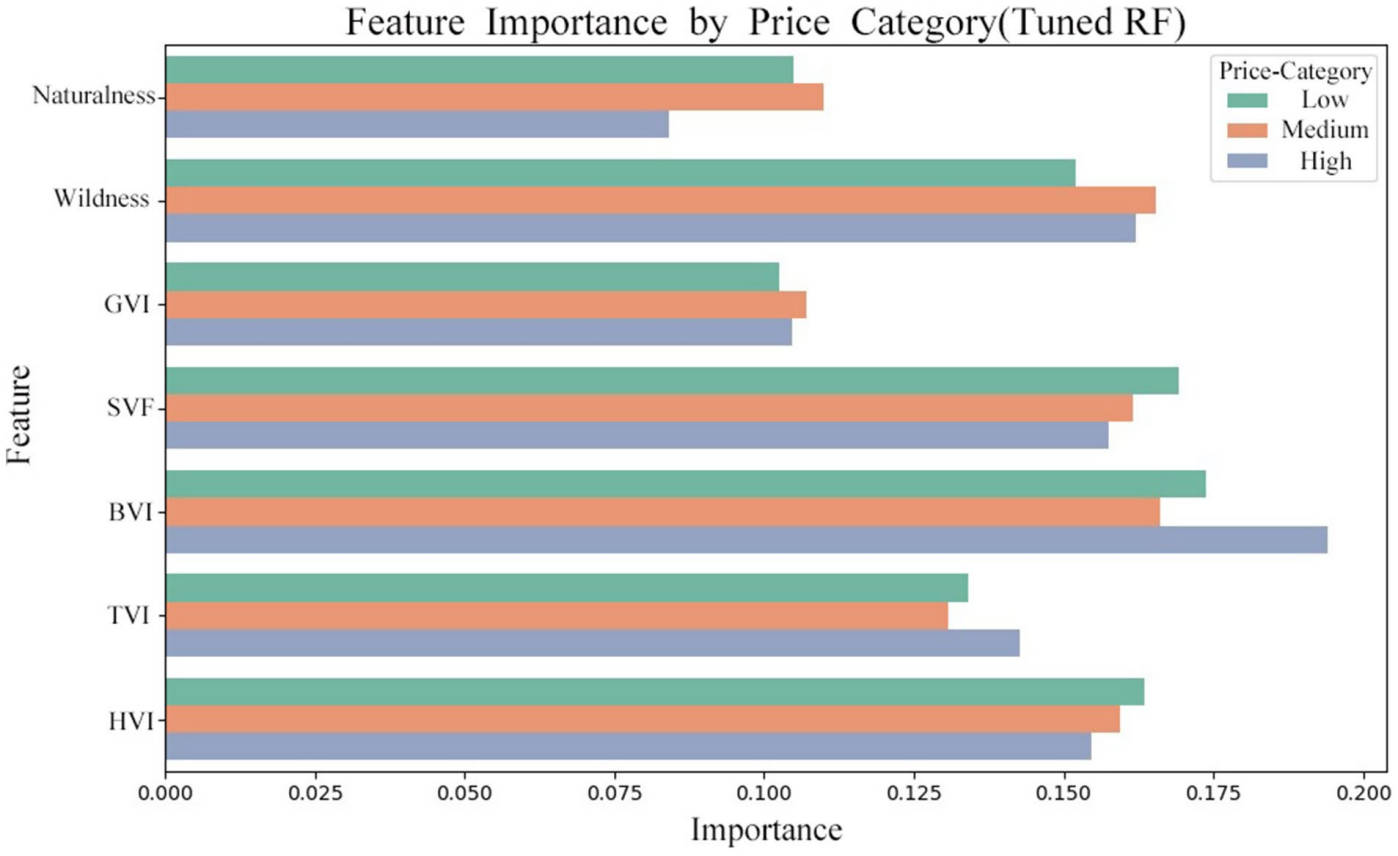
The ranking of the importance of landscape variables under different housing price levels.

Figure 5 indicates that the importance of street-view visual landscape indicators in housing price prediction varies across housing-price groups. Overall, non-natural indicators contribute slightly more than natural indicators. Among all indicators, SVF shows relatively high importance across the low-, medium-, and high-price groups, suggesting that streetscape openness is a stable factor associated with housing prices across different price levels. By contrast, BVI is most important in the high-price group, while Wildness contributes more in the medium- and high-price groups, indicating that the visual environmental factors associated with housing prices differ across socioeconomic strata. Meanwhile, the more pronounced changes in the importance of Naturalness and BVI between the medium- and high-price groups further suggest that the relationship between visual landscape characteristics and housing prices is heterogeneous rather than uniform across housing-price categories.

### 4.4 Partial dependence relationships between visual landscape indicators and housing price predictions

#### 4.4.1 Marginal effects of landscape indicators in low-housing-price areas

**Figure 6:**
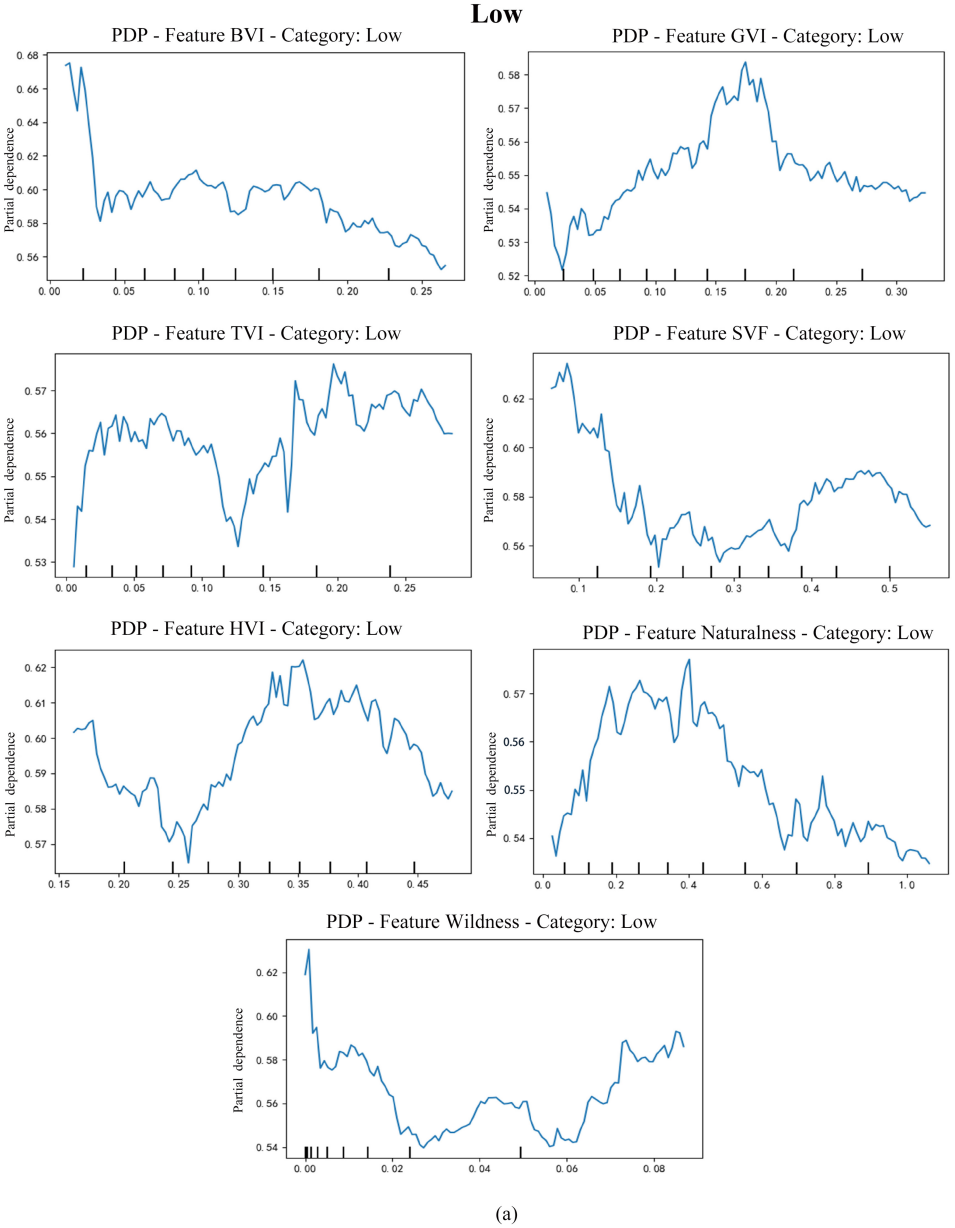
Partial dependence plots of landscape indicators for housing price classification in low-housing-price areas.

Figure 6 shows that, in low-housing-price residential areas, increases in street-view visual indicators within relatively low value ranges generally lead to marked positive changes; however, once certain thresholds are exceeded, the marginal effects gradually weaken, and some indicators even exhibit negative effects. Specifically, the classification probability associated with TVI rises rapidly within the range of 0.00–0.05, then fluctuates and declines at higher values. GVI exhibits a pronounced inverted U-shaped relationship, reaching its peak within the range of 0.15–0.25, while the classification probability remains relatively low at both lower and higher value intervals. This suggests that, in low-housing-price neighborhoods, a moderate increase in tree visibility and visible greenery helps improve shading, alleviate street monotony, and enhance environmental comfort. However, excessively high levels may weaken these positive effects because overly dense tree canopies, visual obstruction, and insufficient daylight may increase the sense of enclosure and reduce perceived safety.

SVF corresponds to a relatively high classification probability at low values, which then declines as SVF increases, indicating that greater openness in such areas does not necessarily correspond to high-quality open space. Instead, it may reflect excessively exposed streets or insufficient shelter, thereby reducing the efficiency of space use. BVI declines rapidly within the range of 0–0.05 and then continues to decrease more gradually, indicating that a higher proportion of building interface generally reduces the classification probability in low-housing-price areas. This may be related to a stronger sense of spatial oppression, as well as problems such as aging buildings, disordered facades, and inadequate ventilation and daylighting. HVI, by contrast, shows a pattern of initial increase followed by decline, rising continuously within the range of 0.25–0.35 and then decreasing slightly. This suggests that a moderate level of human-activity interface helps enhance street vitality and daily convenience, whereas once its proportion becomes too high, its positive effect may be offset by excessive hard paving, intensified environmental disturbance, and increased crowding.

#### 4.4.2 Marginal effects of landscape indicators in medium-housing-price areas

**Figure 7:**
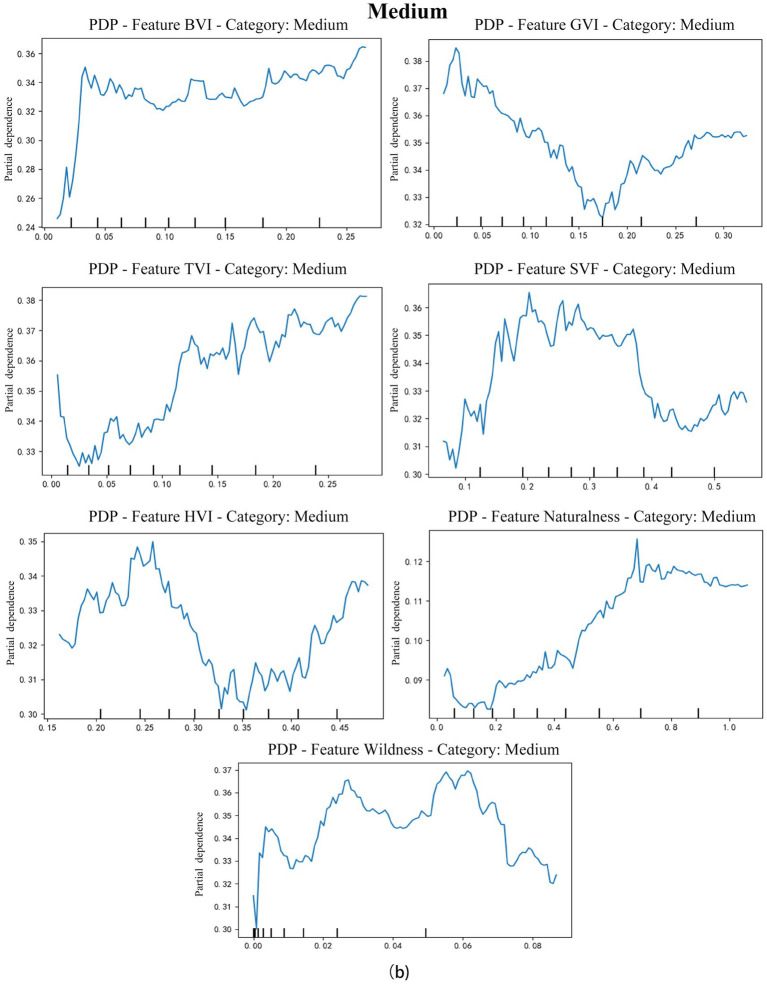
Partial dependence plots of landscape indicators for housing price classification in medium-housing-price areas.

Figure 7 shows that the classification probability associated with TVI increases continuously within the range of 0.05–0.25, indicating a relatively stable positive effect of trees in medium-housing-price residential areas. This may be because trees not only improve shading and landscape quality, but are also commonly found along better-maintained streets. GVI exhibits a pattern of initial decline followed by recovery: the classification probability decreases to approximately 0.32 within the range of 0–0.15, then rises over the interval of 0.15–0.30 and stabilizes at around 0.36. This suggests that visible greenery begins to exert a more evident positive effect only after reaching a certain threshold, after which the marginal effect gradually weakens. A possible explanation is that fragmented greenery is insufficient to effectively improve the street environment, whereas a certain degree of continuity is required before visible greenery can more consistently enhance visual comfort and residential perception.

SVF increases within the range of 0.10–0.30 and reaches a maximum value close to 0.36, indicating that medium-housing-price residential areas exhibit a relatively clear response to spatial openness. Lighting conditions, ventilation, and spatial spaciousness appear to play a more stable role at this price level, which may be related to the greater emphasis placed on everyday residential comfort in medium-housing-price neighborhoods. Naturalness rises significantly over the interval of 0.40–0.70 and then tends to stabilize, suggesting that residents in medium-housing-price areas show a relatively clear preference for environmental naturalness; however, once a certain level is reached, its incremental value no longer continues to expand. BVI increases rapidly within the range of 0–0.05 and then remains within a narrow fluctuation range of 0.34–0.35, indicating that a moderate building interface helps form continuous street space and functional frontage, whereas further increases in its proportion provide only limited additional benefits to environmental quality.

HVI shows a pattern of initial decline followed by recovery, decreasing continuously over the range of 0.15–0.35 and reaching a low point at around 0.30, before rising slowly over the interval of 0.35–0.45 and gradually stabilizing. This indicates that the response of medium-housing-price residential areas to human-activity interfaces is stage-dependent: at low to moderate levels, such interfaces are more likely to reflect traffic disturbance, increased hard paving, or spatial crowding; at higher levels, however, if they correspond to more mature commercial amenities and greater street vitality, their negative effects may be partially alleviated. Wildness exhibits relatively pronounced fluctuations, with a local peak of approximately 0.37 within the range of 0.02–0.07. This suggests that perceptions of “wild” environmental characteristics in medium-housing-price residential areas are not stable, and their effects depend largely on whether natural elements are interpreted as indicators of ecological quality or as signs of insufficient management.

#### 4.4.3 Marginal effects of landscape indicators in high-housing-price areas

**Figure 8:**
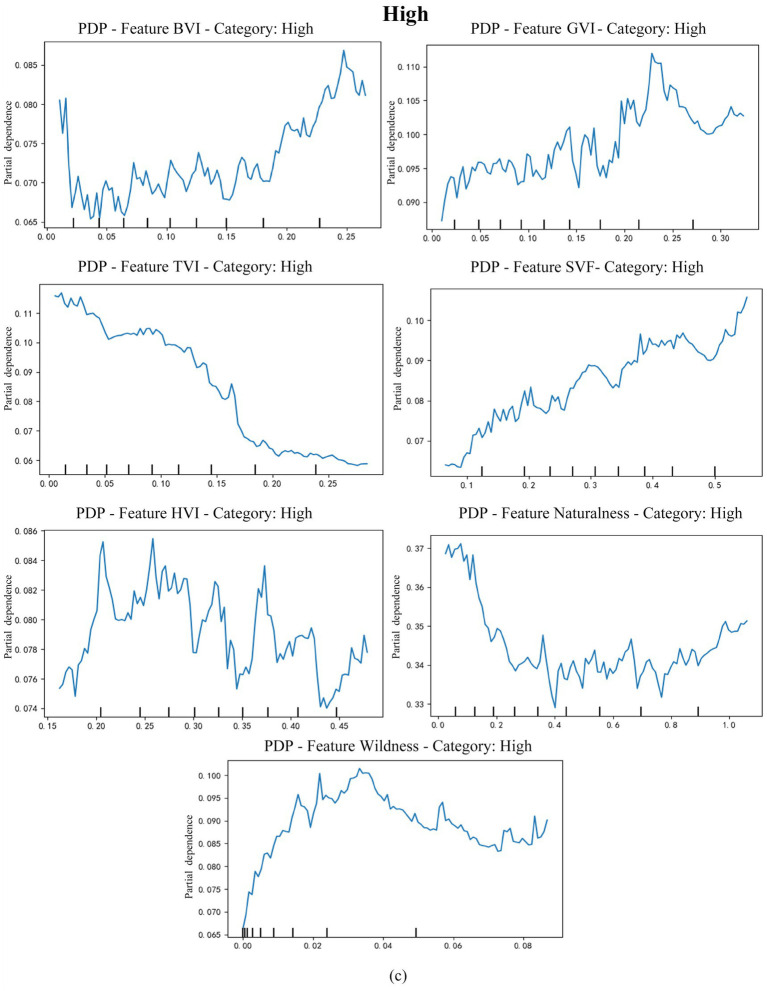
Partial dependence plots of landscape indicators for housing price classification in high-housing-price areas.

Figure 8 shows that as TVI increases from 0 to approximately 0.15, the classification probability declines from about 0.11 to 0.08, and then decreases further to a value close to the minimum (approximately 0.06) as TVI continues to rise to around 0.25. This indicates that greater tree-canopy visibility does not consistently correspond to a higher classification probability. A possible explanation is that high-housing-price residential areas place greater emphasis on daylight access and spatial transparency, and excessive tree-canopy coverage may introduce visual obstruction and a stronger sense of enclosure, thereby weakening its positive effect.

GVI exhibits a relatively pronounced positive effect. Within the range of 0.10–0.25, the classification probability increases from approximately 0.095 to a peak of about 0.11, and then tends to stabilize beyond 0.25. This suggests that visible greenery exerts a relatively stable positive influence in high-housing-price residential areas, although its marginal effect gradually levels off after reaching a certain threshold. This finding indicates that high-housing-price neighborhoods maintain a stable preference for green visual environments, while additional greenery contributes only limited further improvements in environmental quality.

As SVF increases from 0.10 to above 0.50, the classification probability rises from approximately 0.07 to about 0.10, with the slope becoming steeper in the higher-value range. This may be because high-housing-price residential areas place greater value on open views, making higher spatial openness more likely to translate into residential environmental advantages. BVI shows a weak positive relationship within the range of 0.15–0.25, with the classification probability increasing from approximately 0.07 to 0.085. This indicates that, in some high-housing-price neighborhoods, a stronger building interface may correspond to a higher classification probability, although the relationship remains relatively weak overall. A possible explanation is that building interfaces in high-housing-price areas more often reflect enhanced spatial order, but their influence is not dominant compared with green visual environments and spatial openness.

Wildness increases rapidly within the range of 0–0.03, from approximately 0.065 to 0.10, and then declines slightly before stabilizing. By contrast, Naturalness decreases from approximately 0.37 to 0.33 over the interval of 0–0.40 and then rises to about 0.35 within the range of 0.40–1.00. The former suggests that high-housing-price residential areas may derive landscape diversity benefits from a limited degree of naturalness-related wild elements, whereas excessive “wildness” may not align with the preference for refined maintenance and spatial order typically associated with high-end residential environments. The latter indicates that the effect of “naturalness” depends on the extent to which it is compatible with spatial order and environmental maintenance. HVI exhibits relatively frequent fluctuations over the range of 0.15–0.45, with a peak value of approximately 0.085, suggesting that its influence may be jointly shaped by commercial land-use types and neighborhood spatial structure. A moderate increase in HVI may enhance convenience and sense of place, whereas excessive exposure may also lead to increased noise and reduced privacy.

### 4.5 Contribution of visual landscape indicators to the housing price classification model

**Figure 9:**
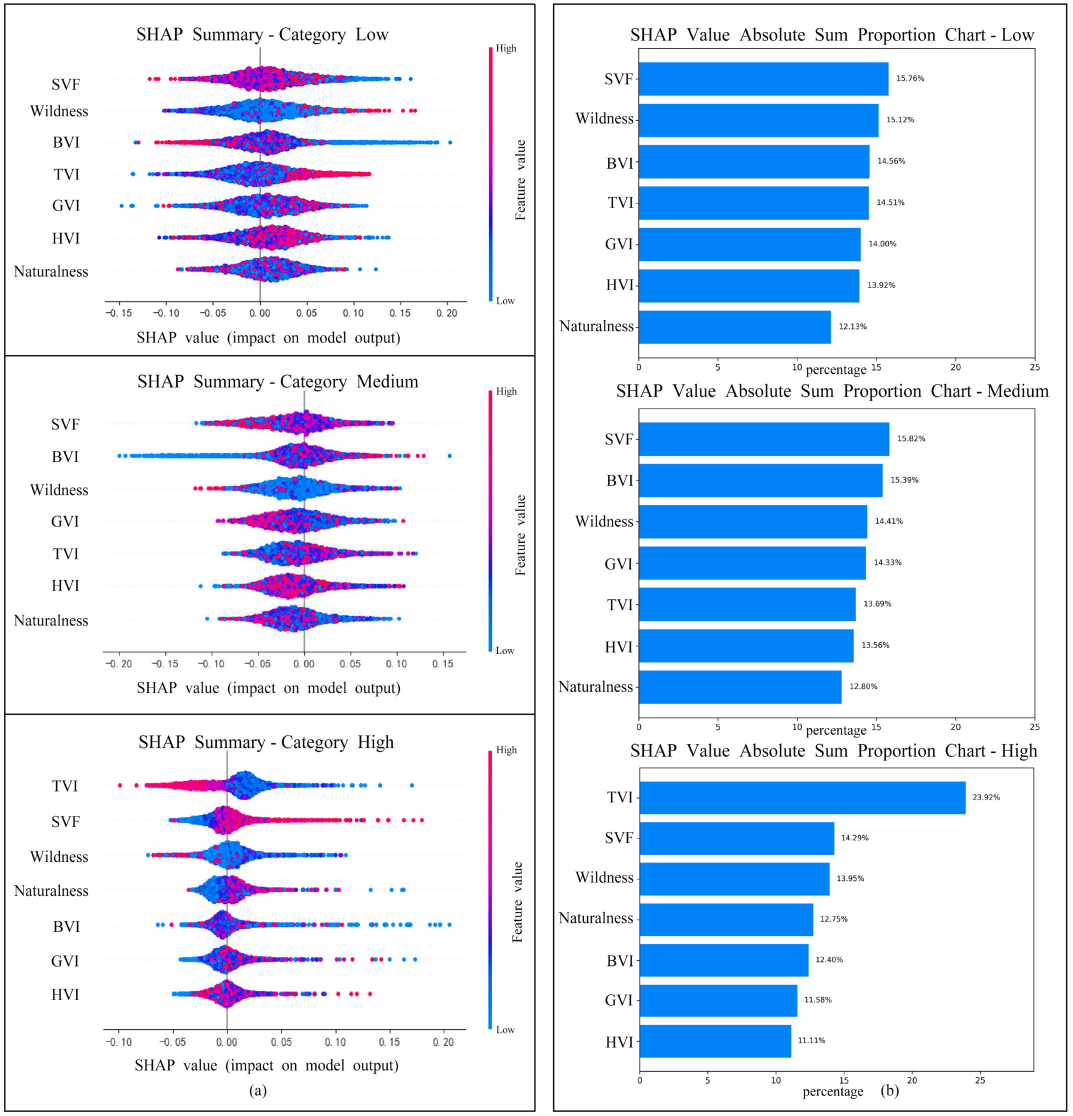
SHAP analysis of landscape indicators across different housing price levels. (a) SHAP value distributions; (b) relative importance of landscape indicators.

In Figure 9, in the low-housing-price group, TVI and GVI show more negative contributions at higher feature values, whereas SVF, Wildness, and BVI exhibit relatively wider SHAP distributions. Indicator importance is generally similar, with SVF ranking highest (15.76%), followed by Wildness (15.12%) and BVI (14.56%), and no single dominant factor emerging, suggesting that the identification of low-priced housing relies on the combined effects of multiple environmental and built-environment characteristics.

In the medium-housing-price group, SHAP distributions are more concentrated overall, with SVF, TVI, and GVI showing clearer positive patterns. SVF contributes the most (15.82%), followed by BVI (15.39%), indicating that medium-priced housing is more consistently associated with environments characterized by greater openness, better daylighting and ventilation, and more mature street interfaces, with spatial comfort playing a broader explanatory role.

In the high-housing-price group, TVI shows the widest SHAP distribution and more concentrated positive contributions, while SVF also maintains a relatively high positive density at higher feature values. The most notable change is the sharp increase in TVI importance to 23.92%, substantially higher than in the low-price (14.51%) and medium-price (13.69%) groups, whereas SVF accounts for 14.29%, indicating that the identification of high-priced housing depends more strongly on visual greening characteristics such as tree-canopy visibility, while also emphasizing spatial openness and landscape quality.

Overall, SVF remains highly important across all three housing-price groups, indicating that spatial openness is a stable explanatory factor across price levels. By contrast, TVI becomes markedly more important in the high-housing-price group, whereas Wildness varies little and Naturalness shows relatively weak independent explanatory power. These findings suggest that streetscape optimization should not follow a uniform standard across housing-price levels: low-price areas should prioritize overall environmental quality improvement, medium-price areas should emphasize openness and comfort, and high-price areas should focus more on tree-canopy landscapes and human-scale ecological quality.

## 5 Discussion

### 5.1 Revisiting green space equity from a human-scale perspective

Against the backdrop of the widespread adoption of the “15-min city” framework in contemporary urban development, ensuring equitable access to green space and accurately representing residents’ lived environmental experiences has emerged as a key issue in urban equity research (52, 53). In densely populated cities such as Chengdu, conventional remote sensing methods often overlook individual-level differences in perceived greenery, thereby underestimating spatial inequality in green space exposure.

To address this gap, this study combines the “15-min city” framework with a human-scale perspective and employs visual landscape indicators to capture visual exposure and subjective environmental experience along residents’ daily mobility paths. In doing so, it extends this framework to green equity research and provides urban policymakers with a resident-centered perspective for green infrastructure planning, thereby responding directly to the practical demands of people-oriented urban governance.

Compared with traditional remote sensing data, greenery perception indicators extracted from street-view images are closer to the greening exposure that residents can actually see and perceive at the street level. Remote sensing indicators mainly characterize the physical coverage and spatial distribution of green spaces, whereas residents’ daily exposure usually occurs along travel routes and street interfaces, and is influenced by factors such as visual obstruction, street cross-sectional form, and tree canopy morphology. Based on this, this study employs indicators such as SVF, GVI, and TVI to characterize, from a human-eye perspective, openness of the field of view, visible greenery, and tree-canopy exposure, thereby better reflecting the actual perceptual differences experienced during walking. This indicator system also provides a scale more closely aligned with behavioral experience for explaining the differences identified in this study in the contributions of landscape factors across different housing price levels.

Findings from Chengdu’s central districts validate the utility of this approach. High-housing-price neighborhoods generally showed higher GVI and TVI values, indicating richer visual experiences and layered vegetation. In contrast, low-price areas often lacked visible greenery, suffered sky obstruction, and exhibited monotony. Despite recorded green space coverage in remote sensing data, these areas frequently lacked perceptual accessibility.

By introducing visual landscape indicators, this study contributes to a conceptual shift in green equity research—from physical accessibility to subjective perceptibility. It highlights the role of visual exposure and perception during daily movement. In high-density settings, building-induced obstruction can make existing greenery imperceptible. The use of GVI, TVI, and SVF uncovers perceptual inequalities overlooked by traditional metrics. Within the “15-min city” framework, this study emphasizes whether greenery can be seen and felt, not just whether it exists—offering a human-centered path for inclusive green planning and social equity advancement.

### 5.2 Mechanisms linking streetscape perceptual features to green space equity

To further reveal the spatial manifestation of inequality, this study examined the response characteristics and mechanisms of streetscape perception indicators across three socioeconomically stratified zones in Chengdu, defined by housing price levels.

In low-price areas, green-related indicators had limited influence. SHAP analysis showed GVI and TVI contributed 14.00 and 14.51%, respectively. Low TVI values were mainly associated with positive SHAP effects, while the GVI PDP curve peaked at 0.58 before declining. This suggests that although green spaces offer shading and cooling, excessive or unmanaged greenery—such as overgrown lots or farmland near urban villages—often lacks planning and maintenance. Under Chengdu’s humid climate, such spaces become visually chaotic and functionally underutilized, limiting perception and engagement. This aligns with Luo, Zhang, Kong, and Dai (54), who noted “ineffective greening” in low-income areas. TVI showed an overall positive trend but dropped sharply between 0.10–0.15, possibly due to tree canopies aligning with windows, causing visual obstruction and confinement. BVI contributed 14.56%, with its PDP curve declining past 0.15, likely reflecting dense older neighborhoods where narrow inter-building distances obstruct green visibility. SVF had the highest contribution (15.76%), with negative SHAP values and a downward-sloping PDP curve, possibly corresponding to open but low-quality environments like urban fringes or inner-city villages characterized by weak design, unclear boundaries, and exposure to vacant or noisy surroundings.

In medium-price areas, TVI contributed 13.69%. Its PDP curve was negative below 0.05 and generally positive above, indicating that ecological benefits of greenery emerge only when TVI exceeds a certain threshold. GVI (14.33%) showed a threshold effect: below 0.17, it had negative impacts, while above it, effects became positive. Low-GVI environments often consist of scattered or unmanaged vegetation lacking structure, while higher values reflect continuous, functional green landscapes (55). SVF had the highest contribution (15.82%), with a PDP curve peaking at 0.2–0.3 and declining beyond. This range suggests an optimal balance between sunlight and shading, improving ventilation and lighting without causing heat stress. Wildness (14.41%) showed mostly negative SHAP effects, with the PDP curve declining sharply beyond 0.06. This may reflect middle-income residents’ sensitivity to unmanaged greenery, where excessive naturalness leads to negative perceptions rather than restorative effects.

In high-price areas, TVI had the highest contribution (23.92%), with high values linked to negative SHAP effects. Its PDP curve peaked at 0.12 and then declined, possibly due to tall walls and hedges in gated communities blocking visibility and openness. This reflects high-income groups’ preference for private, high-quality green spaces. SVF (14.29%) showed consistent positive SHAP values, with increasing marginal benefits when SVF exceeded 0.3. Values between 0.1–0.3 often correspond to CBDs with blocked sky views, while values above 0.3 are found in low-density villa areas with open views and sunlight. This supports Chen et al. (56), who identified “visual openness” as key to luxury housing premiums. GVI (11.58%) rose sharply beyond 0.15, peaking near 0.25, indicating that high-price areas integrate greenery without obstructing visibility, balancing esthetics and ecological function. Naturalness (12.75%) showed a U-shaped PDP curve, with a sharp decline in the 0–0.3 range. Low values are typical in CBDs, while moderate values often indicate poorly maintained greenery. This “high GVI but low Naturalness” pattern suggests a high-income preference for well-maintained, designed green spaces over wild vegetation, echoing Liao, Deng, and Huang (57), who found preferences for lawn-style, low-canopy green areas that avoid oppressive or insecure atmospheres caused by dense tree cover.

### 5.3 Policy implications

This study identifies nonlinear responses of street-view visual perception indicators across different socioeconomic contexts, particularly in the uneven distribution of green visibility and spatial openness across the city. These perceptual inequalities reflect structural inequities in green space distribution and highlight the need for differentiated urban green governance strategies. In low-housing-price areas, especially older neighborhoods, the coordinated renewal of buildings and green spaces should be promoted, with simultaneous optimization of street greenery and building layout to avoid excessive visual obstruction that may reduce ventilation, daylight access, and perceived safety. Green space governance should also be strengthened in peri-urban and underdeveloped areas to improve spatial continuity and equity. In medium-housing-price areas, green space structure should be optimized by balancing greenery intensity with spatial permeability, while refined and graded maintenance systems should be established for public green spaces, with clear standards for pruning, cleanliness, and vegetation replacement. In high-housing-price areas, the public benefits of high-quality green spaces could be moderately extended through approaches such as shared boundary green spaces, allowing limited public access without compromising privacy and thereby helping to reduce the socio-spatial segmentation of green space.

### 5.4 Limitations and prospects

This study developed an analytical framework for evaluating green space equity based on street-view imagery and interpretable machine learning, highlighting green visibility and environmental perception from a human-centered perspective within the “15-min city” framework.

Nonetheless, certain limitations exist. Although housing transaction data offer temporal and spatial detail, they may not capture residential tenure differences, limiting identification of vulnerable populations. Additionally, the update frequency and seasonal variation of street-view images may affect the consistency of indicators such as GVI, TVI, SVF, and Naturalness. Future research can expand by integrating diverse data sources—such as mobility trajectories and social media—to better depict activity spaces and perceptual pathways. Combining longitudinal surveys with psychological perception metrics may also uncover deeper links between green exposure, health, and spatial behavior. Building on this study’s contributions, future work should advance data integration, mechanism modeling, and scenario-based adaptation. As the “15-min city” gains global traction, developing a universally applicable framework for assessing green space equity will become central to sustainable urban planning and environmental justice.

## 6 Conclusion

This study focused on the central urban area of Chengdu and employed spatial statistical analysis and explainable machine learning methods to reveal the spatial differentiation of housing prices and their coupling relationship with visual landscape indicators. The results showed that: (1) housing prices exhibited significant positive spatial clustering and were characterized by a stable core–periphery structure and a local pattern of higher values in the south and lower values in the north; (2) most visual landscape indicators formed significant bivariate high–high (HH) clusters with housing prices in the high-housing-price areas of southern Chengdu, indicating that areas with higher housing prices were usually associated with more pronounced street-view landscape advantages; and (3) the explanatory effects of visual landscape indicators on different housing price levels showed significant stratified differences. Among these indicators, SVF was a stable explanatory variable across all housing price levels, whereas the explanatory effect of TVI was significantly enhanced in the high-housing-price group, suggesting that the high-value housing market was more sensitive to refined greenness characteristics such as visible tree-canopy exposure.

Future research could incorporate locational variables such as education, healthcare accessibility, and rail transit and combine multi-temporal data with causal identification methods to further identify the dynamic relationship between street-view landscapes and housing prices and its underlying mechanisms. Meanwhile, from the perspective of environmental equity, future studies could examine the differential distribution of green visual exposure, spatial openness, and landscape quality across different socioeconomic groups to assess the fairness of the allocation of high-quality environmental resources.

## Data Availability

The original contributions presented in the study are included in the article/Supplementary material, further inquiries can be directed to the corresponding author.

## Footnotes

1
https://ke.com/

2
https://www.tianditu.gov.cn/

3
https://ditu.amap.com/

4
https://map.baidu.com/
