# Supplementary material for: Assessing human-scale green equity in the 15-minute city using street-view-based visual landscape indicators and explainable machine learning: a case study of Chengdu, China
Source: Front Public Health. 2026 Jun 8;14:1847064. doi: 10.3389/fpubh.2026.1847064 (PMC13284084; doi:10.3389/fpubh.2026.1847064)
Supplement: Supplementary file 1 [file Supplementary_file_1.docx]

**Supplementary Material**

**Text S1. Spatial clustering analysis of housing prices**

**S1. Methods**

To examine the spatial clustering of housing prices in the study area, supplementary spatial analyses were conducted using Hot spot analysis (Getis–Ord Gi*), Global Moran’s I, and Local Moran’s I. These methods were used to identify hot and cold spots, assess global spatial autocorrelation, and detect local clusters and spatial outliers.

**Hot Spot Analysis (Getis-Ord** $\boldsymbol{G}_{\boldsymbol{i}}^{\boldsymbol{*}}$**)**

The Getis–Ord Gi* statistic was used to identify significant hot spots and cold spots of housing prices in the study area (Getis & Ord, 1992; Ord & Getis, 1995) . The statistic is calculated as follows:

| $G_{i}^{*}=\frac{\sum_{j=1}^{n} w_{ij}x_{j}-\bar{X}\sum_{j=1}^{n} w_{ij}}{S\sqrt{\frac{n\sum_{j=1}^{n} w_{ij}^{2}-\left( \sum_{j=1}^{n} w_{ij} \right)^{2}}{n-1}}}$ | （1） |
| --- | --- |

where $w_{ij}$denotes the spatial weight between spatial units $i$and $j$, and $x_{j}$represents the housing price of unit $j$. A significantly positive $G_{i}^{*}$value indicates a hot spot, whereas a significantly negative value indicates a cold spot.

**Global Moran’s I**

Global Moran’s I was used to assess the spatial autocorrelation of housing prices across the study area (Moran, 1950). The statistic is calculated as follows:

| $I=\frac{n}{S_{0}}\frac{\sum_{i=1}^{n} \sum_{j=1}^{n} w_{ij}(x_{i}-x)(x_{j}-x)}{\sum_{i=1}^{n} (x_{i}-x)^{2}}$ | （2） |
| --- | --- |

where $S_{0}=\sum_{i=1}^{n} \sum_{j=1}^{n} w_{ij}$. A positive Moran’s I indicates positive spatial autocorrelation, a negative value indicates spatial dispersion, and a value close to zero indicates a random spatial pattern.

**Local Moran’s I**

Local Moran’s I was used to identify local clusters and spatial outliers of housing prices (Anselin, 1995; Ord & Getis, 1995). The statistic is calculated as follows:

| $I_{i}=\frac{\left( x_{i} - x \right)}{m_{2}}\sum_{j=1}^{n} w_{ij}(x_{j}-x)$, $m_{2}=\frac{1}{n}\sum_{k=1}^{n} (x_{k}-x)^{2}$ | （3） |
| --- | --- |

Local spatial association patterns were classified into four types: high–high (HH), low–low (LL), high–low (HL), and low–high (LH). HH and LL indicate local clusters, whereas HL and LH indicate spatial outliers.

**S2. Hot spot and global spatial autocorrelation results of housing prices**

Fig. S1 shows the spatial distribution of hot spots and cold spots of housing prices in the study area. High-value hot spots are mainly concentrated in the southern part of the study area and several central urban districts, particularly Gaoxin District, Wuhou District, and Jinjiang District, whereas low-value cold spots are more frequently found in the northern and peripheral areas, such as parts of Jinniu District and Chenghua District. These patterns indicate marked spatial heterogeneity and clustering of housing prices across the study area.

| 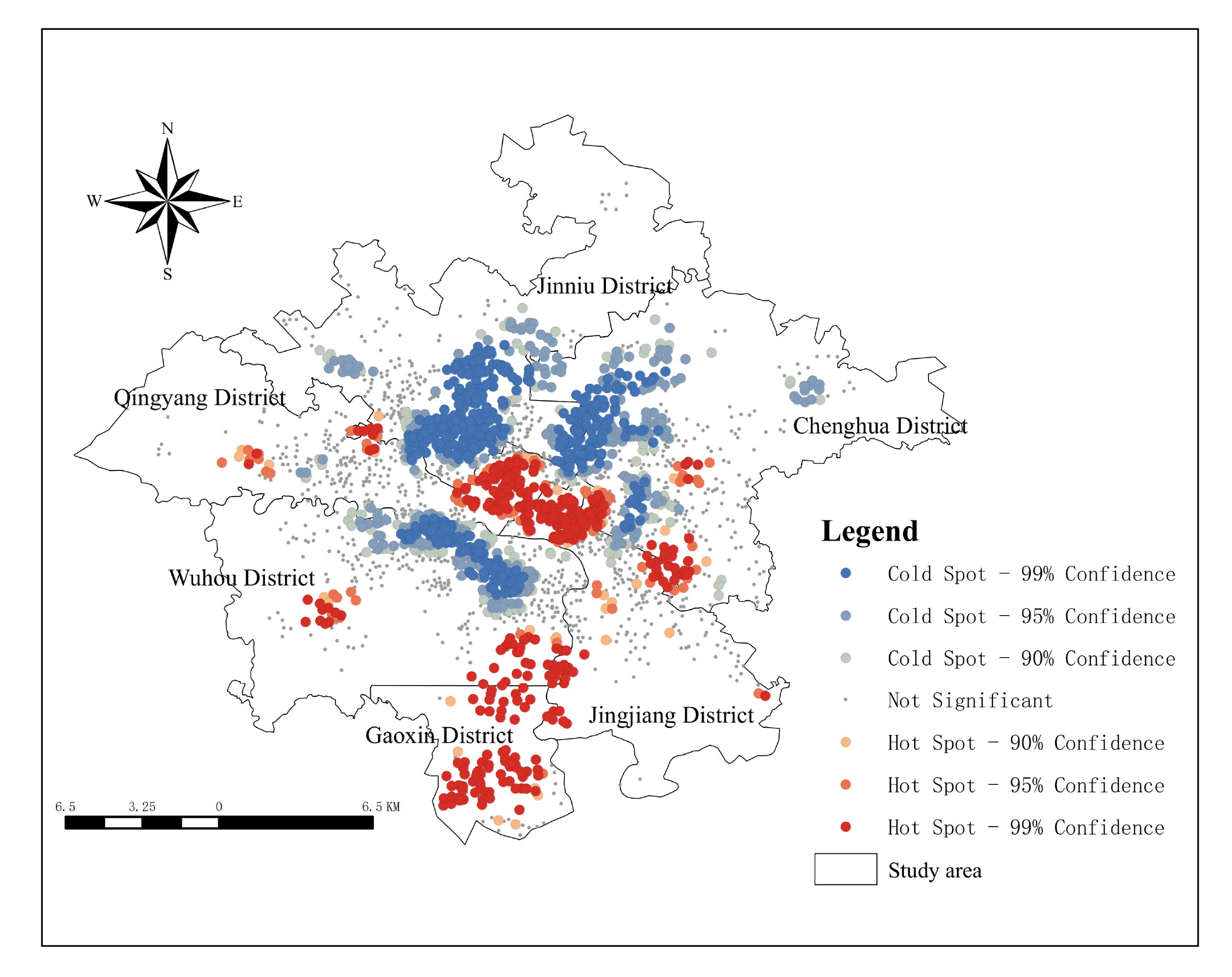 |
| --- |
| **Fig. S1. Hot spot analysis of housing prices in central Chengdu** |

**Table S1. Global Moran’s I statistics for housing prices**

| **Variable** | **Moran’s I** | **p-value** | **Interpretation** |
| --- | --- | --- | --- |
| Housing prices | 0.322 | <0.001 | Significant positive spatial autocorrelation |

The Global Moran’s I result indicates significant positive spatial autocorrelation of housing prices in the study area (Moran’s I = 0.322, p < 0.001), suggesting that housing prices are spatially clustered rather than randomly distributed.

**S3. Local Moran’s I cluster and outlier analysis of housing prices**

Fig. S2 shows the local spatial association patterns of housing prices in the study area. High–high (HH) clusters are mainly concentrated in the southern part of the study area, whereas low–low (LL) clusters are primarily distributed in the northern and some peripheral areas. High–low (HL) and low–high (LH) outliers are relatively limited and scattered. Overall, the Local Moran’s I results further confirm the local clustering characteristics of housing prices and provide a basis for the subsequent analysis of the spatial coupling between housing prices and street-view landscape indicators.


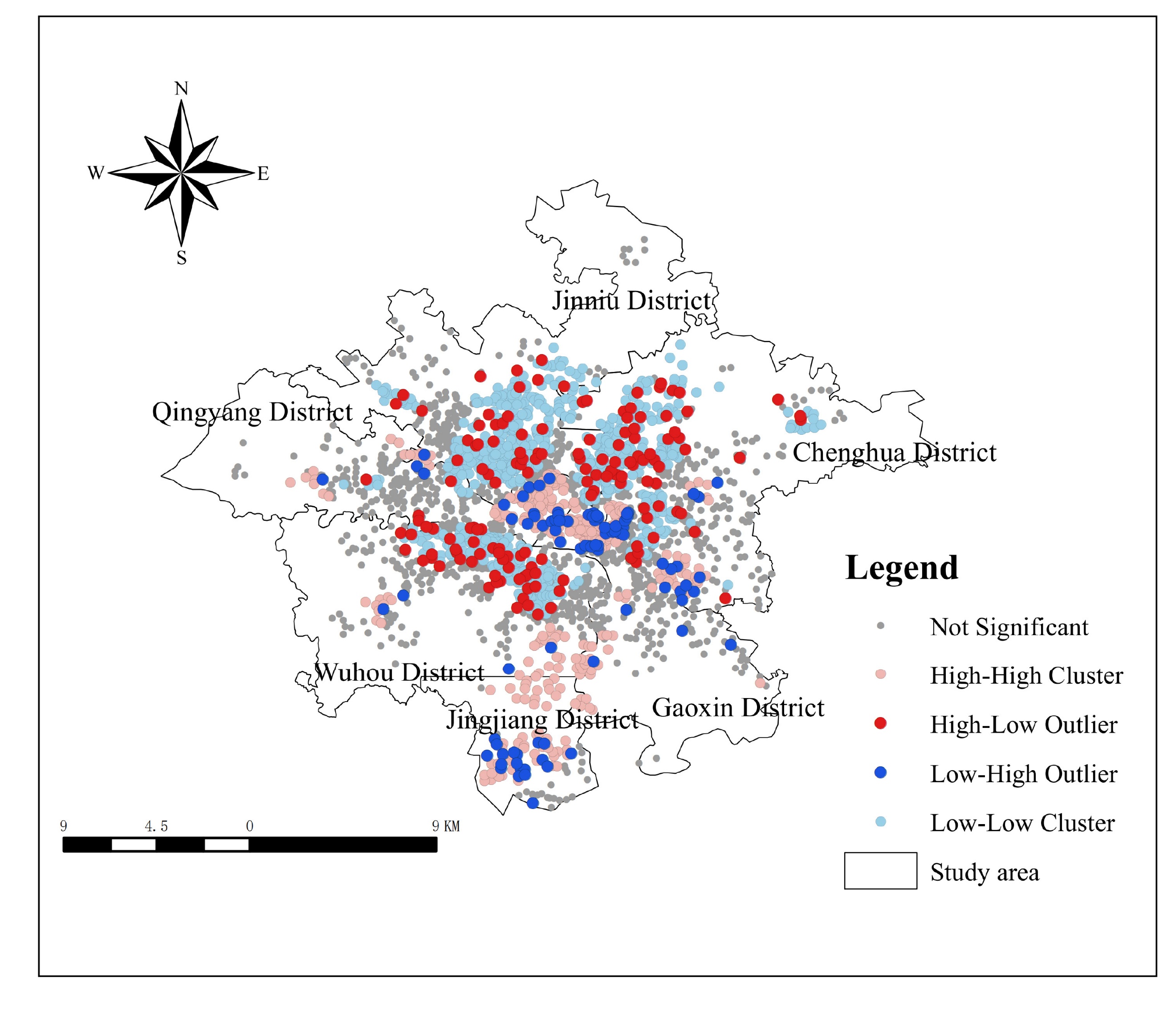


**Fig. S2. Local Moran’s I cluster and outlier map of housing prices in central Chengdu**

**References**

Anselin, L. (1995). Local indicators of spatial association—LISA. *Geographical analysis*, *27*(2), 93-115.

Getis, A., & Ord, J. K. (1992). The analysis of spatial association by use of distance statistics. *Geographical analysis*, *24*(3), 189-206.

Moran, P. A. (1950). Notes on continuous stochastic phenomena. *Biometrika*, *37*(1/2), 17-23.

Ord, J. K., & Getis, A. (1995). Local spatial autocorrelation statistics: distributional issues and an application. *Geographical analysis*, *27*(4), 286-306.
